# Comparative genomic analysis of prion genes

**DOI:** 10.1186/1471-2164-8-1

**Published:** 2007-01-02

**Authors:** Marko Premzl, Vera Gamulin

**Affiliations:** 1 Department of Molecular Biology, Rudjer Boskovic Institute, Zagreb, Croatia

## Abstract

**Background:**

The homologues of human disease genes are expected to contribute to better understanding of physiological and pathogenic processes. We made use of the present availability of vertebrate genomic sequences, and we have conducted the most comprehensive comparative genomic analysis of the prion protein gene *PRNP *and its homologues, shadow of prion protein gene *SPRN *and doppel gene *PRND*, and prion testis-specific gene *PRNT *so far.

**Results:**

While the *SPRN *and *PRNP *homologues are present in all vertebrates, *PRND *is known in tetrapods, and *PRNT *is present in primates. *PRNT *could be viewed as a TE-associated gene. Using human as the base sequence for genomic sequence comparisons (VISTA), we annotated numerous potential *cis*-elements. The conserved regions in *SPRN*s harbour the potential Sp1 sites in promoters (mammals, birds), C-rich intron splicing enhancers and PTB intron splicing silencers in introns (mammals, birds), and hsa-miR-34a sites in 3'-*UTR*s (eutherians). We showed the conserved *PRNP *upstream regions, which may be potential enhancers or silencers (primates, dog). In the *PRNP *3'-*UTR*s, there are conserved cytoplasmic polyadenylation element sites (mammals, birds). The *PRND *core promoters include highly conserved CCAAT, CArG and TATA boxes (mammals). We deduced 42 new protein primary structures, and performed the first phylogenetic analysis of all vertebrate prion genes. Using the protein alignment which included 122 sequences, we constructed the neighbour-joining tree which showed four major clusters, including shadoos, shadoo2s and prion protein-likes (cluster 1), fish prion proteins (cluster 2), tetrapode prion proteins (cluster 3) and doppels (cluster 4). We showed that the entire prion protein conformationally plastic region is well conserved between eutherian prion proteins and shadoos (18–25% identity and 28–34% similarity), and there could be a potential structural compatibility between shadoos and the left-handed parallel beta-helical fold.

**Conclusion:**

It is likely that the conserved genomic elements identified in this analysis represent *bona fide cis*-elements. However, this idea needs to be confirmed by functional assays in transgenic systems.

## 1 Background

The prion diseases are fatal neurodegenerative diseases in humans and animals, which manifest as infectious, inherited and sporadic [1]. The common feature of prion diseases is aberrant metabolism of prion protein PrP. In the cells, PrP may exist as a heterogenous mix of topological isoforms PrP^C ^and may fold into the compact conformation enciphering features of prions PrP^Sc ^[[1-3] and V. R. Lingappa (pers. communication)]. Normal function of PrP is elusive. PrP^C ^may act both pro- and anti-apoptotically, and PrP^Sc ^could have a role in the cellular metabolism as well [[2,4] and V. R. Lingappa (pers. communication)]. Among other phenotypes, PrP could act as a growth factor in the neuronal context [5].

The homologues of human disease genes are expected to contribute to better understanding of physiological and pathogenic processes, and may be regarded as potential drug targets [6]. The first discovered prion protein gene *PRNP *homologue was doppel gene *PRND*, which lies adjacent to *PRNP *in the genomic sequence [7]. It was proposed that *PRND *and *PRNP *arose by an early gene duplication event of an ancestral *PRN *gene. The *PRND*-coded protein doppel Dpl is ≈20–24% identical to PrP and shows the same overall protein architecture but their functions diverged along with their sequences [8] and there is no redundancy between the adult testis-specific Dpl and ubiquitous PrP [9]. The prion protein testis-specific gene *PRNT *is adjacent to *PRND *in the human genomic sequence [10]. It was proposed that *PRNT *may be closer to *PRND *than *PRNP *due to a duplication event that occurred early during eutherian species divergence. However, *PRNT *was not found in mouse, rat and cow [11,12]. The shadow of prion protein gene *SPRN *encoding shadoo Sho was annotated in eutherians and fish [11,13]. Sho is the only known human PrP homologue that contains a conserved middle hydrophobic region.

Comparative genomics is the major strategy for analysis of genomic sequences [6,14-20]. For example, Lee et al. [21] uncovered a large number of conserved noncoding sequences in the syntenic human, mouse and fugu Hox loci. The first comparative genomic analysis of *PRNP *showed non-coding regions conserved between eutherians, as well as that eutherian *PRNP*s have extensively accumulated transposable elements (TE) [22]. Potential cytoplasmic polyadenylation elements (CPE) were annotated in the eutherian and marsupial *PRNP *3'-*UTR*s [23]. *PRNP*, *PRND*, *PRNT *and *SPRN *show similar gene organisations, which encompass two or three exons [7,10,13,22]. However, while the eutherian *PRNP *and *SPRN *promoters incorporate CpG islands, the tissue-specific *PRND *and *PRNT *promoters do not include CpG islands [10,11,22,24]. Furthermore, *PRNP *and *SPRN *are present in both eutherians and fish (the two *PRNP *homologues in fish are *PrP1 *and *PrP2*) but *PRND *was found only in eutherians, and *PRNT *was found only in primates [11,12,25]. Yet, some major differences are known between *PRNP *and *SPRN *[11]. In eutherians, *SPRN *genes are GC-richer and shorter than *PRNP*s and do not harbour TEs. Furthermore, *SPRN*s aligned between human and fish in the long genomic sequence comparisons but not *PRNP*s, and there is contiguity between the adjacent *SPRN *and *GTP *genes conserved between mammals and fish, which was not found for *PRNP*s. One hypothesis has been that the *SPRN *gene evolving more conservatively could be redundant with the less conserved, dispensable *PRNP *[9,11].

We made use of the present availability of vertebrate genomic sequences [20], and we have conducted the most comprehensive comparative genomic analysis of *SPRN*, *PRNP*, *PRND *and *PRNT *so far. We annotated numerous conserved genomic elements which are potential *cis*-elements, deduced 42 new protein primary structures, performed phylogenetic analysis of the prion genes, and showed that the entire PrP conformationally plastic region is conserved between eutherian PrPs and Shos.

## 2 Results and Discussion

### 2.1 Comparative genomic analysis of *SPRN*

The present *SPRN *dataset included 18 genomic sequences, which were from human and 9 eutherians (chimpanzee, rhesus macaque, small-eared galago, mouse, rat, rabbit, cow, dog and little brown bat), 1 marsupial (gray short-tailed opossum), 1 bird (chicken), 1 amphibian (western clawed frog) and 5 fish (fugu, spotted green pufferfish, Japanese medaka, three-spine stickleback and zebrafish).

#### 2.1.1 Conserved contiguity between *SPRN*, *GTP *and *PAOX*

We annotated vertebrate *SPRN *local genomic neighbourhoods using the VISTA tool [26] (not shown), together with the gene predictions from Vega and Ensembl [27] and the *SPRN*-coded cDNAs (Additional data file 1).

**Figure 1:**
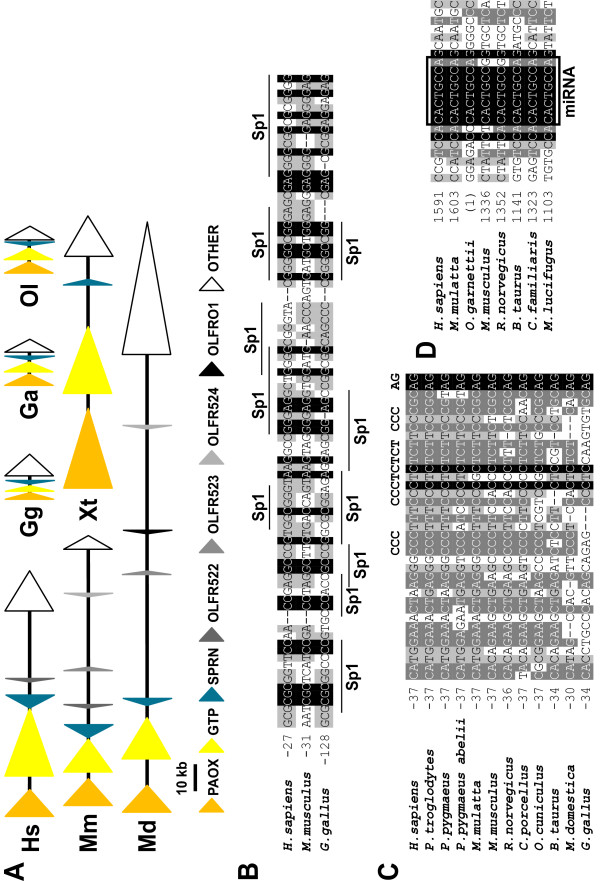
Comparative genomic analysis of *SPRN*. (A) Gene order and relative gene orientations in the local *SPRN *genomic contexts located on the human chr. 10 (Hs), mouse chr. 7 (Mm), gray short-tailed opossum chr. 1 (Md), chicken chr. 6 (Gg), western clawed frog scaffold_502 (Xt), Japanese medaka chr. 15 (Ol) and three-spine stickleback chr. 6 (Ga). Detailed genomic sequence coordinates were given in section 4.1. Gene names were explained in the main text. Genes were drawn approximately to scale. The horizontal bar shows 10 kb sequence length. (B) Conserved region in *SPRN *promoters. Sequence coordinates were calculated relative to introns. Horizontal lines denote predicted Sp1 sites in human (above alignment) and chicken (below alignment). (C) Conserved region in *SPRN *introns. Sequence coordinates were calculated relative to exon 2. CCC, C-rich intron splicing enhancer sequence; CTCTCT, polypyrimidine tract-binding protein-binding site sequence; AG, 3' intron splice site sequence. (D) Conserved motifs in the conserved *SPRN *3'-*UTR *region 7. Sequence coordinates were calculated relative to ORFs where possible. miRNA, potential hsa-miR-34a site (CACTGCCA). (B-D) White letters on black background indicate conservation in 100% sequences, white letters on dark gray background indicate conservation in ≥75% sequences and black letters on light gray background indicate conservation in ≥50% sequences. Ga, *Gasterosteus aculeatus*; Gg, *Gallus gallus*; Hs, *Homo sapiens*; Md, *Monodelphis domestica*; Mm, *Mus musculus*; Ol, *Oryzias latipes*; Xt, *Xenopus tropicalis*.

The contiguity between *SPRN *and distal genes encoding GTP-binding protein (unknown function) *GTP *and peroxisomal amine-oxidase *PAOX *is conserved between vertebrates (Figure 1A), as known for eutherians and pufferfish [11,13]. In western clawed frog, the relative head-to-tail orientation between *SPRN *and *GTP *is different. *Fae *is in place of *paox *in zebrafish [11,13]. These differences may exist due to genomic rearrangements, or due to genomic sequence misassemblies.

On the other hand, genes upstream to *SPRN *differ between vertebrates (Figure 1A). The olfactory receptor 522 pseudogene *OLFR522 *and the scavenger receptor cysteine-rich type 1 protein CD163c-alpha gene *SR *are upstream to *SPRN *in human and chimpanzee, but the *Olfr522*, *Olfr523 *(pseudogene in rat), *Olfr524 *and *Sr *genes are upstream to *Sprn *in mouse and rat. In the present cow genomic assembly, the PWWP domain containing protein gene lies upstream to *SPRN*. In gray short-tailed opossum, the *OLFR523*, opossum-specific gene provisionally termed *OLFRO1*, *OLFR524 *and *SR *genes lie upstream to *SPRN*. The local species-specific expansions of olfactory receptor genes are known in mammals [6,14,16,18,19]. Finally, upstream to *SPRN *are the enoyl-CoA hydratase gene in chicken and in Japanese medaka and three-spine stickleback, the *C20orf29 *homologue in western clawed frog and the vinculin-coding gene in pufferfish.

We also analysed the *SPRNB *genomic contexts in fish. In Japanese medaka and three-spine stickleback, *SPRNB *is located between the calsenilin and *PrP1 *(*stPrP-1*) genes, as known for pufferfish [11,25]. However, we found no *SPRNB *homologue in tetrapods, which suggests that *SPRNB *arose in the fish lineage after the evolutionary separation between fish and tetrapods.

#### 2.1.2 *SPRN*-coded transcripts and SAGE data

In NCBI [28] we found 9 *SPRN*-coded cDNAs, as well as 148 ESTs (Additional data file 1). All cDNAs are from the central nervous system (CNS). The chicken and western clawed frog *SPRN *genes have two exons, as known for eutherians and zebrafish [11,13].

The majority of *SPRN*-coded ESTs is from the CNS (e.g. 20 of 62 human ESTs, 36 of 41 mouse ESTs). Furthermore, the *SPRN *ESTs were found in the immune system cells (mammals and chicken), human kidney and CD34^+^CD38^+ ^stem cells, mouse lung and chicken muscle, as well as in the human tumor EST libraries from the CNS, colon, germ cells, lung, oesophagus, ovary, pancreas, skin, thyroid and uterus, respectively.

The SAGEmap [28] data showed human *SPRN *expression in 6 SAGE and 1 LSAGE libraries from the CNS (Additional data file 2). The majority of evidence for *Sprn *expression from mouse SAGE data is from the CNS libraries (e.g. 8 of 23 SAGE and 7 LSAGE libraries), but there are also evidences  from the CD24+CD25- T cells, forelimb buds, granulosa cells, heart, kidney, skeletal muscle and testis (Additional data file 2).

The new *SPRN *expression evidences, together with the annotation of conserved elements in promoters (section 2.1.3) argue against the initial proposal that *SPRN *expression is highly brain-specific [13], and this discrepancy needs to be resolved experimentally.

#### 2.1.3 Conserved elements in *SPRN *promoters, introns and 3'-*UTRs*

We used VISTA to identify conserved *SPRN *regions, using human as the base sequence in analysis (not shown). Only the coding regions are conserved between human and western clawed frog and fish, but both coding regions and non-coding sequences are conserved between human and chicken and mammals.

The putative *SPRN *promoters contain numerous overlapping Sp1 sites (Figure 1B), which are conserved between human and mouse and chicken. Sp1 typically activates gene expression via GC-rich motifs associated with housekeeping genes and is involved in almost all cellular processes [29]. The associations between promoters, CpG islands and Sp1 sites known for eutherian housekeeping genes, as well as EST and SAGE data (section 2.1.2) suggest that *SPRN*s, like *PRNP*s, may be broadly expressed.

The conserved region in *SPRN *introns includes polypyrimidine tracts and 3' intron splice sites (Figure 1C). Splice sites have relatively low information contents, but not the adjacent intron sequences, which showed elevated substitution rates in comparisons with the synonymous exonic sites [18]. Within the polypyrimidine tracts, we found potential polypyrimidine tract-binding protein PTB-binding sites [30]. PTB is a key splicing repressor in mammals. We also found the potential C-rich intron splicing enhancers [31]. These conserved elements may act as the *SPRN *splicing enhancers or silencers.

In the eutherian *SPRN *3'-*UTR*s, we annotated 11 conserved regions, alignments of which are available on request. Within these conserved regions, we observed numerous highly conserved short motifs. For example, in the region 7 we found 8 bp sequences conserved between human and rhesus macaque, small-eared galago, cow, dog and little brown bat (Figure 1D), which may bind microRNA (miRNA) hsa-miR-34a, as well as the predicted miRNAs MIR141, MIR144 and MIR199 [32]. Similar rat and mouse sequences (Figure 1D) were predicted to bind miRNAs when mismatches were allowed [32]. Therefore, *SPRN *could be a miRNA-regulated gene.

### 2.2 Comparative genomic analysis of *PRNP*, *PRND *and *PRNT*

Our *PRNP*, *PRND *and *PRNT *sample included 25 genomic sequences that were from human and 16 eutherians (chimpanzee, rhesus macaque, small-eared galago, mouse, rat, rabbit, cow, sheep, dog, cat, little brown bat, European shrew, western European hedgehog, nine-banded armadillo, African elephant and small Madagascar hedgehog), 2 marsupials (gray short-tailed opossum and tammar wallaby), 1 bird (chicken), 1 amphibian (western clawed frog) and 4 fish (fugu, spotted green pufferfish, three-spine stickleback and zebrafish).

#### 2.2.1 *PRNP *is present in all vertebrates but not *PRND *or *PRNT*

We used VISTA to annotate genes residing in the vertebrate *PRNP *neighbourhoods, using human as the base sequence in experiments (Additional data file 3), together with the gene predictions from Vega and Ensembl. Genes lying adjacent to *PRNP *in eutherians, pufferfish and zebrafish are known [11,25]. We described for the first time the local *PRNP *genomic neighbourhoods in marsupials, birds, amphibians and three-spined stickleback.

**Figure 2:**
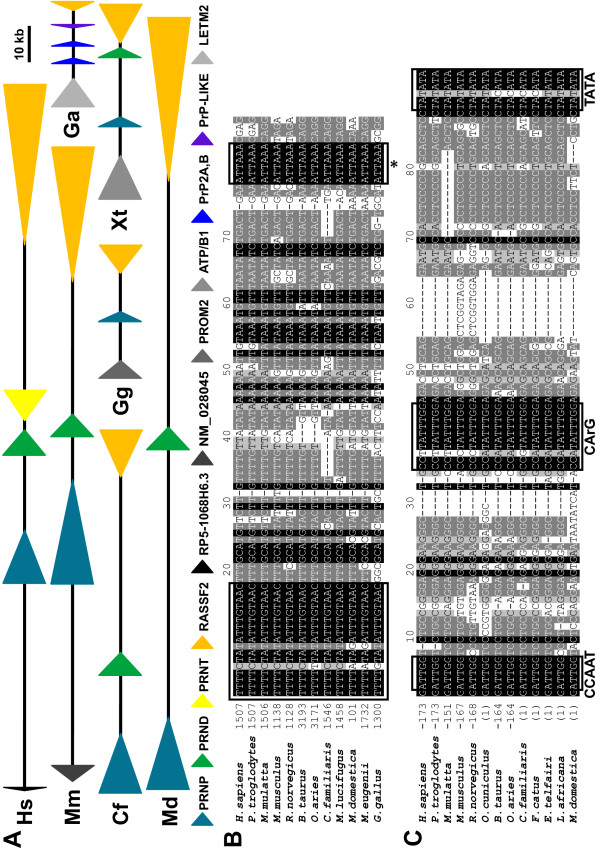
Comparative genomic analysis of *PRNP*, *PRND *and *PRNT*. (A) Gene order and relative gene orientations in the local *PRNP *genomic contexts located on the human chr. 20 (Hs), mouse chr. 2 (Mm), dog chr. 24 (Cf), opossum chr. 1 (Md), chicken chr. 22 (Gg), western clawed frog scaffold_143 (Xt) and three-spine stickleback chr. 13 (Ga). Detailed genomic sequence coordinates were given in section 4.2. Gene names were explained in the main text. Genes were drawn approximately to scale. The horizontal bar shows 10 kb sequence length. (B) Conserved region in *PRNP *3'-*UTR*s. Sequence coordinates were calculated relative to ORFs. Rectangles denote 17 bp elements, which harbour the potential cytoplasmic polyadenylation element (CPE) sequences (TTTTTAT, consensus CPE sequence in sheep) and highly conserved octamers (TTTGTAAC), as well as nuclear specific-polyadenylation signal sites (ATTAAA) labelled by a star. (C) Conserved region in *PRND *promoters. Sequence coordinates were calculated relative to introns where possible. Rectangles denote the conserved CCAAT (ATTGG), CArG (CCTTATTTGG) and TATA (TATATA) boxes. (B, C) White letters on black background indicate conservation in 100% sequences, white letters on dark gray background indicate conservation in ≥75% sequences and black letters on light gray background indicate conservation in ≥50% sequences. Cf, *Canis familiaris*; Ga, *Gasterosteus aculeatus*; Gg, *Gallus gallus*; Hs, *Homo sapiens*; Md, *Monodelphis domestica*; Mm, *Mus musculus*; Xt, *Xenopus tropicalis*.

Genes located upstream to *PRNP *differ between vertebrates (Figure 2A), which includes the human RP5-1068H6.3 pseudogene, NM_028045 in mouse, cow zinc finger protein *ZMYND11 *(not shown), chicken prominin 2 *PROM2*, mitochondrial ATP synthase B chain precursor in western clawed frog *ATP/B1 *and leucine zipper-EF-hand containing transmembrane protein 2 in three-spine stickleback *LETM2*. The *PRNP *gene is present in all tetrapods, and its homologue *PrP2 *(*stPrP-2*) is present in fish [11,25]. Due to the extensive divergence of their sequences [11], human *PRNP *did not align with *PrP2*s (Additional data file 3). The two *PrP2 *homologues are present in three-spine stickleback, here referred to as *PrP2A *and *PrP2B*. Thus there are three *PrP *genes in three-spine stickleback (*PrP1*, *PrP2A *and *PrP2B*). *PrP-like *lies adjacent to *PrP2 *in all fish, but it is not present in tetrapods [11,25]. *PRND *is present in eutherians and marsupials, but we did not detect *PRND *in birds. *PRND *is absent from fish [11,25]. However, in western clawed frog we found a potential ORF encoding a protein which is similar to Dpls (section 2.3.1). Although no ESTs and *ab initio *gene predictions correspond to this ORF, we could not rule out the presence of a *PRND*-like gene in western clawed frog, suggesting that a duplication of an ancestral gene giving rise to *PRNP *and *PRND *occurred after separation between fish and tetrapods [7,11,25]. *PRNT *is present in primates (section 2.2.4) [12]. The Ras association domain family 2 gene *RASSF2* is present in all vertebrates.

Therefore, among the prion genes, only *SPRN *and *PRNP *are present in both fish and tetrapods.

#### 2.2.2 Conserved regions in *PRNP *promoters, introns and 3'*UTRs*

Using VISTA comparisons, we identified 7 conserved regions in the *PRNP *upstream intergenic regions, 5 conserved regions in the provisional *PRNP *promoters, 15 conserved regions in the *PRNP *introns and 5 conserved regions in the *PRNP *3'-*UTR*s (alignments are available on request). Some of these regions were already described [22,23], and we focused here on the most interesting annotations.

The prominent intergenic region lying ≈-12/-7 kb upstream to human *PRNP *is conserved between human and chimpanzee and dog (Additional data file 3). These sequences showed no matches to ESTs or known genes, and they exceed more stringent conservation criteria for detection of intergenic regulatory elements (>70% identity per 100 bp [21]). The sizes of conserved intergenic regions, their conservation levels, as well as their relative distances from *PRNP*s could suggest that they may regulate *PRNP *expression as enhancers or silencers. The shorter aligned regions in rabbit and little brown bat also exceed the more stringent conservation criteria.

One region in *PRNP *3'-*UTR*s is conserved between human and mammals and birds (Figure 2B and Additional data file 3). This region includes highly conserved nuclear polyadenylation signals, and the 17 bp elements, which include the potential CPEs [23] and perfectly conserved 8 bp motifs abundant in human and mouse, rat and dog 3'-*UTR*s [32]. Indeed, *PRNP *was annotated as a likely CPE-specific RNA binding protein substrate in rat [33], and PrP is involved in the development of neuronal polarity *in vitro *[5].

#### 2.2.3 Conserved regions in *PRND *promoters, introns and 3'*UTRs*

Using VISTA comparisons, we identified 25 conserved regions in the intergenic sequences between *PRNP*s and *PRND*s, 7 conserved regions in the *PRND *provisional promoters, 1 conserved region in the *PRND *exon 1s, 5 conserved regions in the *PRND *introns, and 8 conserved regions in the *PRND *3'-*UTR*s (alignments are available on request). We showed the most interesting annotations.

The *PRND *core promoter region [24] is conserved between human and mammals, and it includes highly conserved CCAAT, CArG and TATA elements (Figure 2C). *PRND *has an unclear mode of expression that is developmentally regulated [7,10,24]. The CCAAT boxes are the most critical activator of *PRND *expression in mouse and cow [24]. Our analysis suggests that the conserved CArG boxes binding serum responsive factor may be involved in regulation of *PRND *expression.

In the *PRND *3'-*UTR*s we found the TTGCAATA octamers (lying 2634–2641 bp distally to the human *PRND *ORF), which are conserved between primates, dog and little brown bat. The elements were predicted to bind the annotated miRNAs called MIR45, MIR166 and MIR216 [32].

#### 2.2.4 *PRNT *is a TE-associated gene

The comparative analyses showed that *PRNT *is absent from mouse, rat, cow and fish [11,12]. The present VISTA plot showed extensive sequence conservation between human *PRNT *and chimpanzee and rhesus macaque (Additional data file 3). We compared the human *PRNT *sequence with the eutherian genomic sequences lying between *PRND *and *RASSF2 *(Additional data file 4), and annotated the *PRNT *ORFs from chimpanzee, Sumatran orang-utan and rhesus macaque [EMBL:BN000890, EMBL:BN000891, EMBL:BN000892]. Choi et al. also reported functional *PRNT *ORFs in primates [12]. However, no *PRNT*-coded ORFs were found in the other eutherians. The human *PRNT*-coded protein we called Prt is 93, 95 and 87% identical to the chimpanzee, Sumatran orang-utan and rhesus macaque Prts, respectively (Additional data file 4). No signal peptides were predicted for Prts, which suggests that Prts are intracellular proteins. Our attempts to align Prts with either Dpls or PrPs were not successful.

TEs correspond to ≈35% of human *PRNT *(Additional data file 4). These elements in primates, rabbit, cow, dog and African elephant (but not in mouse and rat) aligned with their human homologues. The processed pseudogene RP51068H6.1 is present only in primates. The discernable interspersed repetitive sequences comprise the majority of mammalian genomes, and they may be resurrected as new genes [6,14,16,18,19]. TEs may acquire coding potential [34] and regulatory functions in promoters, 5'-*UTR*s and 3'-*UTR*s [35]. Thus the *PRNT *exons could have been partially recruited from TEs. For example, the sense LINE2 in human *PRNT *ORF may have acquired a coding function. Accordingly, *PRNT *could be viewed as a TE-associated gene.

### 2.3 Phylogenetic analysis of prion genes

From the available genomic sequences, cDNAs and ESTs [20,27,28], we deduced a total of 39 new protein primary structures, including 15 Shos which were from chimpanzee [EMBL:BN000837], Sumatran orang-utan [EMBL:BN000846], rhesus macaque [EMBL:BN000842], white-tufted-ear marmoset [EMBL:BN001004], rabbit [EMBL:BN000843], domestic guinea pig [EMBL:BN000844], cow [EMBL:BN000839], dog [EMBL:BN000838], little brown bat [EMBL:BN001003], gray short-tailed opossum [EMBL:BN000840], chicken [EMBL:BN000836], western clawed frog [EMBL:BN000841], Japanese medaka [EMBL:BN001007], three-spine stickleback [EMBL:BN000845] and fathead minnow [EMBL:BN001008], 2 Sho2s which were from Japanese medaka [EMBL:BN001013] and three-spine stickleback [EMBL:BN001005], 1 PrP-like which was from three-spine stickleback [EMBL:BN001006], PrP2A and PrP2B which were from three-spine stickleback [EMBL:BN001010, EMBL:BN001011], 2 PrP1s which were from Japanese medaka [EMBL:BN001012] and three-spine stickleback [EMBL:BN001009], 7 PrPs which were from Sumatran orang-utan [EMBL:BN000848], thirteen-lined ground squirrel [EMBL:BN000993], little brown bat [EMBL:BN000992], large flying fox [EMBL:BN000994], zebra finch (2 alleles) [EMBL:BN000995, EMBL:BN000996] and western clawed frog [EMBL:BN000849], 10 Dpls which were from rhesus macaque [EMBL:BN000886], white-tufted-ear marmoset [EMBL:BN001002], horse [EMBL:BN000997], bottle-nosed dolphin [EMBL:BN001001], western European hedgehog [EMBL:BN000998], little brown bat [EMBL:BN001000], African elephant [EMBL:BN000999], small Madagascar hedgehog [EMBL:BN000889], Hoffmann's two-fingered sloth [EMBL:BN000991] and gray short-tailed opossum [EMBL:BN000887]. We aligned these sequences with the 6 Shos, 4 Sho2s, 3 PrP-likes, 4 PrP2s, 7 PrP1s, 47 PrPs and 12 Dpls, as well with the potential western clawed frog Dpl (a total of 123 proteins, the alignment is available on request), and performed phylogenetic analysis.

#### 2.3.1 Phylogenetic tree of prion genes

**Figure 3:**
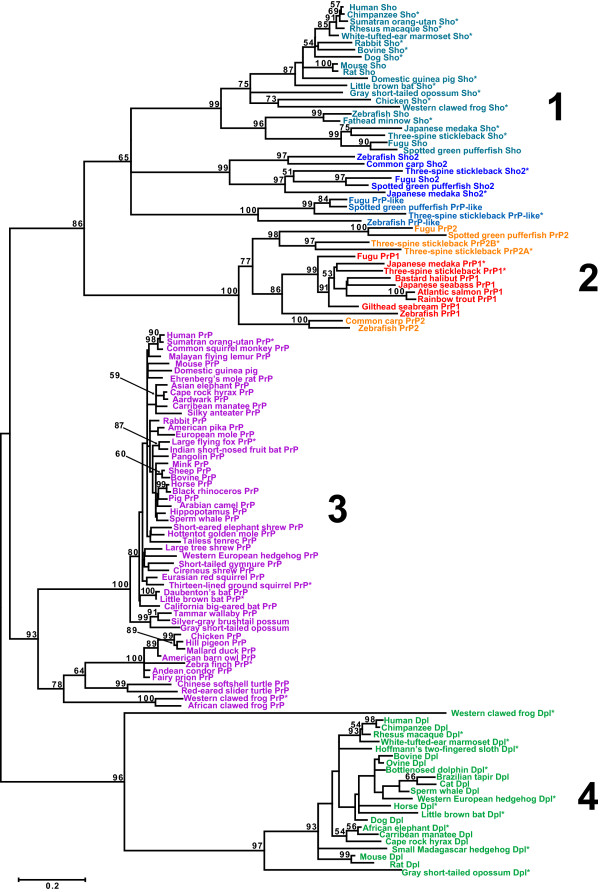
The bootstrap consensus NJ tree for prion genes (122 proteins, 5000 replicates). The bootstrap values >50 are depicted. The horizontal bar shows 0.2 amino acid substitutions per site. 1–4, major clusters.

Using the neighbour joining (NJ) method, we constructed the first phylogenetic tree including all prion genes (Figure 3). The protein tree topology shows four major clusters. The first major cluster includes Shos, Sho2s and PrP-likes. Cotto et al. [36] also noted the clustering of Shos and PrP-likes (PrP3s) in a separate cluster from PrP1s and PrP2s. The tetrapode and fish Shos grouped in the two separate groups [11]. There is a discrepancy between the grouping of the biased sample of mammalian Shos and the species tree topology [37], which needs to be re-examined with additional sequences. The second major cluster comprises the fish PrP1s and PrP2s, which together with the grouping within the cluster agree with the previous analyses [11,25,36]. The pattern suggests that the subfunctionalization of PrP1s and PrP2s may have occurred [11] after a whole genome duplication in the fish lineage [11,21,25,36]. The third major cluster includes the tetrapode PrPs. The mammalian PrPs are positioned on the separate branch. The grouping of the eutherian PrPs is discordant with the species tree topology, as already known for the PrP protein trees [38-41]. The PrPs from birds and reptiles grouped in the two separate groups, which lie on the branch separate from amphibian PrPs. The fourth major cluster includes Dpls. The more distant western clawed frog Dpl is an outgroup to the mammalian Dpls, whose grouping is discordant with the species tree topology and needs to be re-examined with additional species. Our phylogenetic analysis complements analyses of vertebrate prion genes [11,23,25,36,38-45].

#### 2.3.2 PrP plastic region is well conserved in Shos

**Figure 4:**
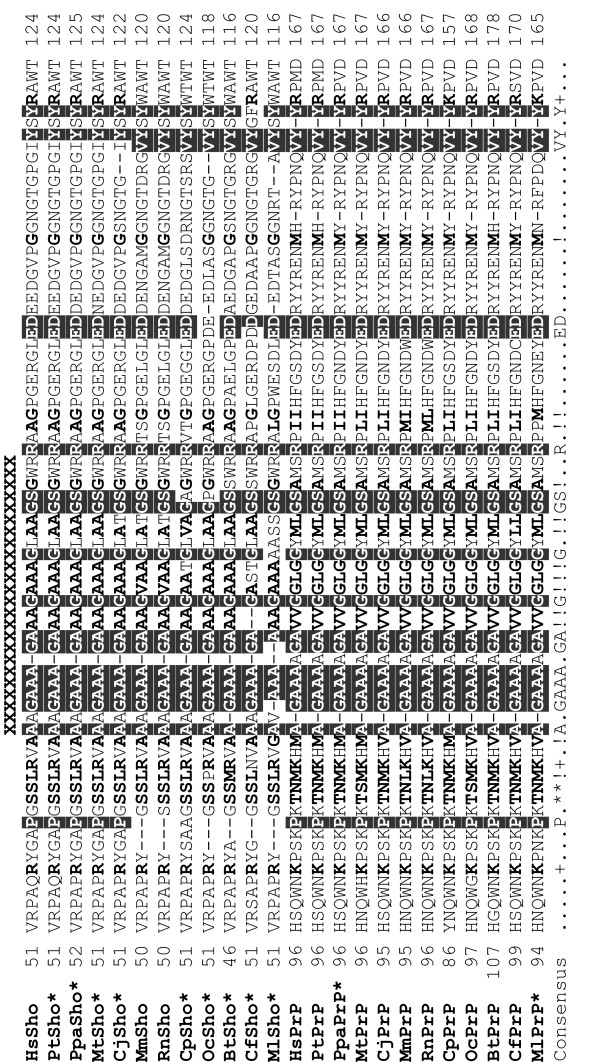
The conserved plastic PrP region compared with Shos. White letters on black background, conserved amino acids; bold, similar amino acids. X indicates residue in the highly conserved potential transmembrane region. In the consensus line: capital letters, conserved amino acids; +, conserved basic residues; *, conserved polar residues; !, conserved hydrophobic residues. Bt, *Bos taurus*; Cf, *Canis familiaris*; Cj, *Callithrix jacchus*; Cp, *Cavia porcellus*; Hs, *Homo sapiens*; Mm, *Mus musculus*; Mt, *Macaca mulatta*; Oc, *Oryctolagus cuniculus*; Ppa, *Pongo pygmaeus abelii*; Pt, *Pan troglodytes*; Rn, *Rattus norvegicus*. *, sequence annotated in this study.

The present Sho dataset enabled us to better define the extent of sequence conservation between PrPs and Shos. Along the entire PrP conformationally plastic region [3], there is 18–25% identity and 28–34% similarity between eutherian PrPs and Shos (Figure 4). Therefore, any functional and structural similarity that may exist between PrPs and Shos resides within the PrP plastic region. The best conserved stretch of plastic region between PrPs and Shos is the PrP transmembrane region (TM), which together with its adjacent basic sequence (stop transfer effector sequence) regulates the choice of PrP topology at the endoplasmic reticulum [3]. The conserved potential TM region sequences in Shos, as well as their basic adjacent sequences could suggest that a choice of Sho topology may be regulated.

We threaded the conserved sequences from several Shos onto the left-handed parallel β-helical sequence 3D profile (Table 1). There is a sensible fit of Sho primary structures to the 3D profile, which comprises three rungs and one short loop. The rung 1 and rung 3 core volumes are more similar to an average of 335 Å than those of rung 2, but similar differences were also observed for the PrP rung 2 core volumes [3]. The rung 3 L3' and L5' arginines, as well as L3" glutamic acid residues may be tolerated [3]. This threading suggests a potential structural compatibility between Shos and the left-handed parallel β-helical fold.

## 3 Conclusion

It is likely that the conserved genomic elements identified in this analysis represent *bona fide cis*-elements. However, this idea needs to be confirmed by functional assays in transgenic systems.

## 4 Methods

### 4.1 Comparative genomic analysis of *SPRN*

We used the public genomic sequences harbouring *SPRN *and adjacent genes from human (VEGA:10:135081619:135169358 from VEGA), chimpanzee (CHIMP2.1:10:134664772:134840875 from Ensembl), rhesus macaque (MMUL_0_1:SCAFFOLD5188:1:85000 from Ensembl, which did not include genes upstream to *SPRN*), small eared galago (BUSHBABY1:scaffold_119777:1:24499 from Ensembl, which included only *SPRN*), mouse (NCBIM36:7:139977004:140082456 from Ensembl), rat (RGSC3.4:1:199910407:200064500 from Ensembl), rabbit (RABBIT:GeneScaffold_3980:20000:43893 from Ensembl, which did not include genes upstream to *SPRN*), cow (Btau_3.1:26:46850000:46960000 from Ensembl), dog ([GenBank:NW_140397] and the overlapping traces TI277811272, TI310201176 and TI296043878 from Trace Archive [28], which did not include genes upstream to *SPRN*), little brown bat (MICROBAT1:scaffold_139987:1:184283 from Ensembl, which did not include genes upstream to *SPRN*), gray short-tailed opossum (BROADO3:1:562720743:563122104 from Ensembl), chicken (WASHUC2:6:10486826:10506320 from Ensembl), western clawed frog (JGI4.1:scaffold_502:494972:606518 from Ensembl), fugu (FUGU4:scaffold_24: 866033:880929), spotted green pufferfish (TETRAODON7:17:4621488:4636712 from Ensembl), Japanese medaka (MEDAKA1:15:23173000:23189443, as well as the *SPRNB*-including sequence MEDAKA1:12:13875000:13908107 from Ensembl; the data has been provided freely by the National Institute of Genetics and the University of Tokyo for use in this publication only), three-spine stickleback (BROADS1:groupVI:8045112:8059132, as well as the *SPRNB*-including sequence BROADS1:groupXIV:6825668:6855018 from Ensembl) and zebrafish (ZFISH6:13:25836077:25841469 from Ensembl, which included only *sprn*). In the sequences, TEs were masked using the slow speed RepeatMasker mode [46]. We used the AVID alignment program implemented in VISTA to compare human or mouse (base sequence) with the other 17 species, respectively. The empirically determined cutoffs for detection of conserved regions were: 95% identity between human and chimpanzee in 100 bp windows, 90% identity between human and rhesus macaque in 100 bp windows, 70% identity between human and small-eared galago in 100 bp windows, 85% identity between mouse and rat in 90 bp windows, 60% identity between base sequence and the other eutherians in 70 bp windows, 55% identity between base sequence and the marsupial gray short-tailed opossum in 60 bp windows, 50% identity between base sequence and chicken and western clawed frog, respectively, in 60 bp windows and 50% identity between base sequence and fish in 50 bp windows. Using fish *SPRNB*s as BLAST queries, we searched the available tetrapode genomes in Ensembl.

The Human_EST, Mouse_EST and EST_others EST libraries in NCBI were searched using available *SPRN*s as queries and BLASTN. The human SAGEmap dataset included 327 libraries with 1296360 unique tags and 19300584 total tag counts, and the mouse SAGEmap dataset included 213 libraries with 1552119 unique tags and 16549657 total tag counts. We used *Nla*III and the human *SPRN *cDNA [GenBank:BC040198] (tags CCCCAGGGCA or CCCCAGGGCACTGAGGG) or the mouse *Sprn *cDNA [GenBank:BC056484] (tags ATGAAACTTT or ATGAAACTTTGTCTGAA) as queries. In order to avoid the sequencing error bias, a tag count was accepted only if counted at least twice in a library.

We used VISTA to compare the human *SPRN *gene including 1.1 kb of its upstream genomic sequence (the distance between putative transcription start site and the first upstream TE) with the other 17 *SPRN *genes and their flanking intergenic sequences, which were each extracted from the long genomic sequences described above. We used alignments between human and species other than primates to define the conserved *SPRN *regions. Gene regions conserved above the cutoff values for VISTA were manually extracted, aligned, inspected and edited using BioEdit [47]. Transcription factor-binding sites in conserved sequences were predicted using TESS [48], using the core positions of TRANSFAC strings with the maximum allowable string mismatch 10%, minimum log-likelihood ratio score 12, minimum string length 6 bp and organism classification vertebrata options. Potential *cis*-elements in *SPRN *introns and 3'-*UTR*s were identified manually. The genomic sequences corresponding to the conserved *SPRN *intron region from orang-utan (TI706538521), Sumatran orang-utan (TI873168233, TI872371190 and TI869752121) and domestic guinea pig (TI798862625) were found in Trace Archive.

### 4.2 Comparative genomic analysis of *PRNP*, *PRND *and *PRNT*

We used the public genomic sequences harbouring *PRNP*, *PRND *and *PRNT *from human (VEGA:20:4558073:4938939 from VEGA), chimpanzee (CHIMP2.1:20:4543476:4892558 from Ensembl), rhesus macaque (MMUL_0_1:SCAFFOLD5559:1:71794:1 from Ensembl), small-eared galago (BUSHBABY1:scaffold_100540:1:125000 from Ensembl), mouse (NCBIM36:2:131546857:131836553 from Ensembl), rat (RGSC3.4:3:119614427:119894427 from Ensembl), rabbit (RABBIT:GeneScaffold_2359:500000:745068 from Ensembl), cow (Btau_3.1:13:46581184:46759911 from Ensembl), sheep (the overlapping [GenBank:U67922], [GenBank:AY184242] and [GenBank:AY017311] sequences including only *PRNP *and *PRND*), dog (BROADD2:24:19625473:19880474 from Ensembl), cat (CAT:scaffold_163520:165841:168496 from Ensembl, which included only *PRND*), little brown bat (MICROBAT1:scaffold_165241:1:50000 from Ensembl, which included only *PRNP*; MICROBAT1:scaffold_165240:1:55347 from Ensembl, which included only *PRND*), European shrew (COMMON_SHREW1:scaffold_217921:1:16448 from Ensembl, which included only *PRNP*; COMMON_SHREW1:scaffold_192527:1:20562 from Ensembl, which included only *PRND*), western European hedgehog (HEDGEHOG:scaffold_373527:1:92000 from Ensembl, which included *PRNP *and *PRND*), nine-banded armadillo (ARMA:scaffold_98578:1:9100 from Ensembl, which included only *PRNP*), African elephant (BROADE1:scaffold_6014:1:76073 from Ensembl), small Madagascar hedgehog (TENREC:scaffold_285038:120000:135234 from Ensembl, which included only *PRND*), gray short-tailed opossum (BROADO3:1:562720743:563122104 from Ensembl), tammar wallaby ([GenBank:AY659987], which included only *PRNP*), chicken (WASHUC2:22:422500:460000 from Ensembl), western clawed frog (JGI4.1:scaffold_143:1551715:1633755 from Ensembl), fugu (FUGU4:scaffold_7:2830000:2860000 from Ensembl), spotted green pufferfish (TETRAODON7:12:9564452:9597016 from Ensembl), three-spine stickleback (BROADS1:groupXIII:3987940:4030338 from Ensembl) and zebrafish (ZFISH6:10:19772658:20135698 from Ensembl). We used the VISTA tool to compare human (base sequence) with the other 24 species, respectively, as in section 4.1.

From the long genomic sequences, we extracted the *PRNP *and *PRND *sequences, respectively, together with their adjacent intergenic regions, and compared them using VISTA. The potential transcription factor-binding sites in promoters were predicted using TESS, and the potential *cis*-elements in introns and 3'-*UTR*s were identified manually. We note that some conserved genomic regions were not evident in the VISTA plot using long genomic sequences (Additional data file 3).

Using VISTA, we compared the human *PRNT *gene with the sequences lying between *PRND *and *RASSF2 *from chimpanzee, rhesus macaque, mouse, rat, rabbit, cow, dog and African elephant, respectively (the other eutherians either did not include this region or included gaps in sequences). For VISTA, we used unmasked sequences. The new *PRNT *ORFs were annotated using genomic sequences (Ensembl, Trace Archive), and deposited in EBI as the third party annotations [49]. The TE analyses were performed using RepeatMasker as above.

### 4.3 Phylogenetic analysis of the prion genes

Using the public genomic sequences, as well as ESTs and cDNAs [20,27,28], we identified new *SPRN *(*Pan troglodytes*, *Pongo pygmaeus abelii*, *Macaca mulatta*, *Callithrix jacchus*, *Oryctolagus cuniculus*, *Cavia porcellus*, *Bos taurus*, *Canis familiaris*, *Myotis lucifugus*, *Monodelphis domestica*, *Gallus gallus*, *Xenopus tropicalis*, *Oryzias latipes*, *Gasterosteus aculeatus*, *Pimephales promelas*), *SPRNB *(*Oryzias latipes*, *Gasterosteus aculeatus*), *PrP-like *(*Gasterosteus aculeatus*), *PrP2A *(*Gasterosteus aculeatus*), *PrP2B *(*Gasterosteus aculeatus*), *PrP1 *(*Oryzias latipes*, *Gasterosteus aculeatus*), *PRNP *(*Pongo pygmaeus abelii*, *Spermophilus tridecemlineatus*, *Myotis lucifugus*, *Pteropus vampyrus*, *Taeniopygia guttata*, *Xenopus tropicalis*) and *PRND *(*Macaca mulatta*, *Callithrix jacchus*, *Equus caballus*, *Tursiops truncatus*, *Erinaceus europaeus*, *Myotis lucifugus*, *Loxodonta africana*, *Echinops telfairi*, *Choloepus hoffmanni*, *Monodelphis domestica*) ORFs, and deposited them in EBI as the third party annotations. The western clawed frog Dpl sequence was translated from JGI4.1:scaffold_143:1604545:1605090 (Ensembl). For alignments, we also used the previously annotated Shos (*Homo sapiens *[GenBank:CAG34288], *Mus musculus *[GenBank:CAG34289], *Rattus norvegicus *[GenBank:CAG34290], *Danio rerio *[GenBank:CAD35503], *Takifugu rubripes *[GenBank:CAG34291], *Tetraodon nigroviridis *[GenBank:CAG30521]), Sho2s (*Danio rerio *[GenBank:CAG34293], *Cyprinus carpio *[GenBank:CAG34294], *Takifugu rubripes *[GenBank:CAG34292], *Tetraodon nigroviridis *[GenBank:CAG34295]), PrP-likes (*Takifugu rubripes *[GenBank:BAC01166], *Tetraodon nigroviridis *[translated from TETRAODON7:12:9573812:9574333 from Ensembl], *Danio rerio *[GenBank:NP_991149]), PrP2s (*Takifugu rubripes *[GenBank:AAR99478], *Tetraodon nigroviridis *[GenBank:CAG30664], *Cyprinus carpio *[GenBank:AAQ76701], *Danio rerio *[GenBank:CAG28803]), PrP1s (*Takifugu rubripes *[GenBank:AAN38988], *Paralichthys olivaceus *[GenBank:AAW33660], *Lateolabrax japonicus *[GenBank:AAW33661], *Salmo salar *[GenBank:AAN38989], *Oncorhynchus mykiss *[GenBank:AAO62075], *Sparus aurata *[GenBank:ABB90540], *Danio rerio *[GenBank:CAG28804]), the balanced set of eutherian PrPs [41] and a subset of the other tetrapode PrPs (*Monodelphis domestica *[GenBank:DAA05687], *Trichosurus vulpecular *[GenBank:AAA61833], *Macropus eugenii *[GenBank:AAT68002], *Gallus gallus *[GenBank:NP_990796], *Columba rupestris *[GenBank:AAF73436], *Anas platyrhynchos *[GenBank:AAF82604], *Tyto alba *[GenBank:AAD47049], *Vultur gryphus *[GenBank:AAD47045], *Pachyptila turtur *[GenBank:AAD47050], *Pelodiscus sinensis *[GenBank:BAC66701], *Trachemys scripta *[GenBank:CAB81568], *Xenopus laevis *[GenBank:CAC86159]) and a subset of Dpls (*Homo sapiens *[Swiss-Prot:Q9UKY0], *Pan troglodytes *[GenBank:XP_525256], *Mus musculus *[GenBank:NP_075530], *Rattus norvegicus *[GenBank:XP_230542], *Bos taurus *[GenBank:NP_776583], *Ovis aries *[GenBank:NP_001009261], *Tapirus terrestris *[GenBank:AAM94875], *Physeter catodon *[GenBank:AAM94877], *Canis familiaris *[GenBank:XP_542905], *Felis catus *[GenBank:AAM94876], *Trichechus manatus *[GenBank:AAM94872], *Procavia capensis *[GenBank:AAM94873]). The protein sequences were aligned using the ClustalW program implemented in BioEdit. The alignments were inspected and manually corrected, and they include both complete and incomplete sequences. We used MEGA3 [50] to infer the NJ phylogenetic tree, using the pairwise deletion option and Poisson correction distance. Only one new zebra finch PrP allele was used for the phylogenetic analysis [EMBL:BN000995], so that the NJ tree includes 122 sequences.

We threaded the potential Sho plastic region sequences onto the left-handed parallel β-helical sequence 3D profile [3]. The starting point for threading was the sequence of mouse PrP β-helical rung 2 region (residues 110–125), which is highly conserved in Shos. A complete triangular left-handed β-helical rung includes 6 different positions repeated three times giving a total of 18 amino acids. The more conserved positions in the 3D profile are interior-facing L3, L5, L3', L5', L3" and L5" restricted to small hydrophobic residues and threonine and serine. The core rung volume was calculated as the sum of side-chain volumes of interior residues for each complete rung. Side-chain volumes were calculated by subtracting the Van der Waals volume of glycine from the Van der Waals volume of an amino acid [3].

## Authors' contributions

MP designed and carried out the studies and drafted the manuscript. VG participated in the design of the study and approved the manuscript.

## Supplementary Material

Additional data file 1 *SPRN*-coded transcripts (ESTs, cDNAs) Click here for file

Additional data file 2 Human and mouse *SPRN *expression data from SAGEmap Click here for file

Additional data file 3 Multiple species comparisons of the vertebrate *PRNP *genomic neighbourhoods. The VISTA plot indicates extent of sequence conservation in the pairwise long genomic sequence alignments between human (base sequence) and chimpanzee, rhesus macaque, small-eared galago, mouse, rat, rabbit, cow, sheep, dog, cat, little brown bat, European shrew, western European hedgehog, nine-banded armadillo, African elephant, small Madagascar hedgehog, gray short-tailed opossum, tammar wallaby, chicken, western clawed frog, fugu, spotted green pufferfish, three-spine stickleback and zebrafish, respectively. The human gene order and transcription directions were shown by grey arrows, coding regions were shown by blue rectangles, and untranslated gene regions were denoted by light blue rectangles. Gene names were explained in the main text. Peaks fitting the empirical cutoffs for conservation in coding sequences, untranslated gene regions, and non-exonic regions, respectively, were labelled blue, light blue, and pink, respectively. On y axis, percents of sequence conservation were depicted for each alignment. 1, conserved intergenic regions; 2, conserved *PRNP *3'-*UTR*s; 3, conserved *PRND *promoters. Click here for file

Additional data file 4 *PRNT *analysis. (A) Multiple species comparisons. The VISTA plot indicates extent of sequence conservation in the pairwise long genomic sequence alignments between human (base sequence) and chimpanzee, rhesus macaque, mouse, rat, rabbit, cow, dog and African elephant, respectively. The human gene order and transcription directions were shown by grey arrows, coding regions were shown by blue rectangles, and untranslated gene regions were denoted by light blue rectangles. Gene names were explained in the main text. Peaks fitting the empirical cutoffs for conservation in coding sequences, untranslated gene regions, and non-exonic regions, respectively, were labelled blue, light blue, and pink, respectively. On y axis, percents of sequence conservation were depicted for each alignment. TE order and relative orientations in the human sequence were shown by triangles. Rectangles denote TEs in *PRNT *exons and aligned sequences. Alu, Alu transposable element; CNS, conserved non-exonic sequence; DNA, DNA transposon fossil; LTR, long terminal repeat of endogenous retrovirus ERV3; L2, LINE2 fossil; UTR, untranslated gene region. (B) Primate Prts. White letters on black background, residues conserved in 100% sequences; white letters on dark grey background, residues conserved in 75% sequences; black letters on light grey background, residues conserved in 50% sequences. Hs, *Homo sapiens *[GenBank:CAD20691]; Mt, *Macaca mulatta*.  Ppa, *Pongo pygmaeus abelii*; Pt, *Pan troglodytes*;  *, sequence annotated in this study. Click here for file

## Figures and Tables

**Table 1:** Threading of Sho primary structures onto the left-handed parallel β-helical sequence 3D profile

Species (residues in Sho)	Rung 1. Human, r. macaque, mouse, rat, dog: V = 234Å^3^; bovine: V = 215Å^3^																	
	
	L1	L2	L3	L4	L5	L6	L1'	L2'	L3'	L4'	L5'	L6'	L1"	L2"	L3"	L4"	L5"	L6"
Human (41–65)	G	G	**A**	R	**G**	A	S	R	**V**	R	**V**	X^a^	X^a^	G	**S**	S	**L**	R
R. macaque (41–65)	G	G	**A**	R	**G**	T	S	R	**V**	R	**V**	X^a^	X^a^	G	**S**	S	**L**	R
Mouse (40–61)	G	G	**A**	R	**G**	A	S	R	**V**	R	**V**	X^a^	X^a^	G	**S**	S	**L**	R
Rat (40–61)	G	G	**A**	R	**G**	A	S	R	**V**	R	**V**	X^a^	X^a^	S	**S**	S	**L**	R
Bovine (37–58)	-	G	**G**	R	**G**	A	A	R	**V**	R	**V**	X^a^	X^a^	G	**S**	S	**M**	R
Dog (41–63)	G	G	**A**	R	**G**	A	P	R	**V**	R	**V**	X^a^	X^a^	G	**S**	S	**L**	N

Species (residues in Sho)	Rung 2. Human, r. macaque, bovine: V = 114Å^3^; mouse, rat: V = 152Å^3^; dog: V = 121Å^3^																	
	
	L1	L2	L3	L4	L5	L6	L1'	L2'	L3'	L4'	L5'	L6'	L1"	L2"	L3"	L4"	L5"	L6"

Human (66–82)	-	V	**A**	A	**A**	G	A	A	**A**	G	**A**	A	A	G	**A**	A	**A**	G
R. macaque (66–82)	-	V	**A**	A	**A**	G	A	A	**A**	G	**A**	A	A	G	**A**	A	**A**	G
Mouse (62–78)	-	V	**A**	A	**A**	G	A	A	**A**	G	**A**	A	A	G	**V**	A	**A**	G
Rat (62–78)	-	V	**A**	A	**A**	G	A	A	**A**	G	**A**	A	A	G	**V**	A	**A**	G
Bovine (59–74)	-	V	**A**	A	**G**	A	-	A	**A**	G	**A**	A	A	G	**A**	A	**A**	G
Dog (64–78)	-	V	**A**	A	**A**	G	-	A	**A**	A	**G**	A	-	G	**A**	S	**T**	G

Species (residues in Sho)	Rung 3. Human, r. macaque: V = 224Å^3^; mouse, rat: V = 256Å^3^; bovine: V = 258Å^3^; dog: V = 234Å^3^																	
	
	L1	L2	L3	L4	L5	L6	L1'	L2'	L3'	L4'	L5'	L6'	L1"	L2"	L3"	L4"	L5"	L6"

Human (83–100)	L	A	**A**	G	**S**	G	W	R	**R**	A	**A**	G	P	G	**E**	R	**G**	L
R. macaque (83–100)	L	A	**A**	G	**S**	G	W	R	**R**	A	**A**	G	P	G	**E**	R	**G**	L
Mouse (79–96)	L	A	**T**	G	**S**	G	W	R	**R**	T	**S**	G	P	G	**E**	L	**G**	L
Rat (79–96)	L	A	**T**	G	**S**	G	W	R	**R**	T	**S**	G	P	G	**E**	L	**G**	L
Bovine (75–91)	L	A	**A**	G	**S**	S	W	R	**R**	A	**A**	G	-	P	**A**	E	**L**	G
Dog (79–93)	-	L	**A**	A	**G**	S	-	S	**W**	R	**R**	A	-	P	**G**	L	**G**	E

Bold, constrained interior-facing rung positions L3, L5, L3', L5', L3" and L5"; V, core rung volume; X^a^, loop in human Sho (52 RPAQRYGAP 60), rhesus macaque Sho (52 RPAPRYGAP 60), mouse Sho (51 RPAPRY 56), rat Sho (51 RPAPRY 56), bovine Sho (47 RPAPRY 53) and dog Sho (52 RSAPRYG 58).
