# Supplementary material for: Comparative genomic analysis of prion genes
Source: BMC Genomics. 2007 Jan 2;8:1. doi: 10.1186/1471-2164-8-1 (PMC1781936; doi:10.1186/1471-2164-8-1)
Supplement: Additional data file 1 — SPRN-coded transcripts (ESTs, cDNAs) [file 1471-2164-8-1-S1.doc]

### Additional data file 1: *SPRN*-coded transcripts (ESTs, cDNAs)

| Species | Tissue | GenBank accession number |
| --- | --- | --- |
| Human | CD34^+^CD38^+^ cell | AI312329, AW075103 |
|  | CNS | AA350041, AK123191 ^a^, BC040198 ^a^, BF946647, BG702476, BI915830, BM714035, BM728076, BM929809, BQ880113, BU737672, BY796386, CA336064, CD580523, DB302994, DN990499, F11557, F13341, R20569, R60457, T77092, Z42653 |
|  | CNS, oligodendroglioma | AI345428 |
|  | Colon, tumor | AI445100, AW954555 |
|  | Germ cell, tumor | AI634039, AI824952, AI991700, AW590109, BE552292 |
|  | Kidney | AW293555 |
|  | Lung, carcinoma | AI310773, AI310842, AI311199, AI311279, AI311506, AI311518, AI311900, AI335080, AI344070, AI344242, AI344249, AI345933, AI371575, AI377150, AI377604, AI378701, AW615822 |
|  | Lung, cystic fibrosis | BM982297 |
|  | Oesophagus, carcinoma | AI355435 |
|  | Ovary, tumor | AA477470, AA477598, AI246918, AW271134, BU844922 ^b^ |
|  | Pancreas, carcinoma | BE906320 |
|  | Skin, melanoma | BF690134 |
|  | Spleen | BQ709341 |
|  | T cell | BX388069 ^b^ |
|  | Thyroid, carcinoma | AW874152 |
|  | Uterus, carcinoma | AI690747 |
|  | (Unknown) | BE707484 |
| Mouse | CNS | AI842512, AI847827, AK049995 ^a^, AU256101, AW061273, BB284188, BB431842, BB653489, BC056484 ^a^, BE647927, BE861218, BE861390, BE989852, BE989935, BF458649, BM933564, BM942367, BI729617, BM944750, BQ174215, BU609729, BU700177, BY261932, BY373511, BY605863, BY613717, BY619969, BY621760, BY648198, BY713524, CA315182,CD354566, CJ069838, CJ146771, CJ158685, CJ249881, CJ253961, DW712185 |
|  | Embryo | AA114743, AI426937, AI550081 |
|  | Lung | BB812286 |
|  | (Unknown) | CR520469 |
| Rat | CNS | AA943106, AI007831, AI101223 |
|  | Mixed tissue | BF285497, BF285504, AW530092, BF391059, BF404416, BF409274, BF409392, BF409521, BF564096, BG376848 |
| Bovine | CNS | DV828917 |
| Dog | Thymus | DN354037 |
| Chicken | CNS | BU391991, CR390504 ^a^ |
|  | Muscle | BM487041 |
|  | T cell | AW240138 |
| Western clawed frog | CNS | CX836955 ^a^ |
|  | Embryo | CX364487 |
| Japanese medaka | Embryo | AM299462 |
| Three spine stickleback | CNS | DN714324, DN714325, DN720225, DN720226, DN729156, DN729157, DW673448, DV000570, DN690460 |
|  | Embryo | DW630748 |
| Fathead minnow | CNS | DT226300 |
| Zebrafish | CNS | AJ490525 ^a^, BC094981 ^a^, BG738569, BI671054, CN504291, DT869844, NM_198981 ^a^ |
|  | Whole fish, embryo | AL913622, AL913623, AL913624, AL913625, AL925410, CD757090, CK140486, CO927245, CO959244, CV491170 |

^a^, cDNA; ^b^, cell line. CNS, central nervous system.
