# Supplementary material for: Comparative genomic analysis of prion genes
Source: BMC Genomics. 2007 Jan 2;8:1. doi: 10.1186/1471-2164-8-1 (PMC1781936; doi:10.1186/1471-2164-8-1)
Supplement: Additional data file 2 — Human and mouse SPRN expression data from SAGEmap [file 1471-2164-8-1-S2.doc]

## Additional data file 2: Human and mouse *SPRN* expression data from SAGEmap

| Species | Library | GEO accession number | Tissue | tc ^a^ | tpm ^b^ |
| --- | --- | --- | --- | --- | --- |
| Human | SAGE | 1. GSM7498 | CNS, Gaucher disease | 4 | 126 |
|  |  | 2. GSM14799 | CNS | 18 | 58 |
|  |  | 3. GSM763 | CNS | 2 | 31 |
|  |  | 4. GSM786 | CNS, seizure | 2 | 25 |
|  |  | 5. GSM41379 | CNS | 2 | 23 |
|  |  | 6. GSM676 | CNS | 2 | 21 |
|  | LSAGE | 1. GSM31935 | CNS | 16 | 52 |
| Mouse | SAGE | 1. GSM3685 | CD4^+^CD25^-^ T cell, CD3-treated | 3 | 140 |
|  |  | 2. GSM12541 | CNS | 16 | 517 |
|  |  | 3. GSM12540 | CNS | 9 | 288 |
|  |  | 4. GSM12513 | CNS | 2 | 262 |
|  |  | 5. GSM17246 | CNS | 4 | 220 |
|  |  | 6. GSM766 | CNS | 8 | 184 |
|  |  | 7. GSM17430 | CNS | 3 | 182 |
|  |  | 8. GSM2415 | CNS | 2 | 102 |
|  |  | 9. GSM788 | CNS, granule cell precursors, sonic hedgehog-treated | 2 | 23 |
|  |  | 10. GSM55 | Forelimb buds | 2 | 29 |
|  |  | 11. GSM30721 | Granulosa cell, PMSG-treated | 2 | 40 |
|  |  | 12. GSM23548 | Heart, angiotensin II-treated | 10 | 551 |
|  |  | 13. GSM23547 | Heart, saline-treated | 10 | 541 |
|  |  | 14. GSM1681 | Heart | 18 | 213 |
|  |  | 15. GSM15116 | Heart | 2 | 77 |
|  |  | 16. GSM7764 | Heart | 2 | 18 |
|  |  | 17. GSM24255 | Kidney, uranyl nitrate-treated | 12 | 512 |
|  |  | 18. GSM24256 | Kidney | 4 | 314 |
|  |  | 19. GSM38207 | Skeletal muscle, adrenalectomized mice | 20 | 143 |
|  |  | 20. GSM38112 | Skeletal muscle | 5 | 84 |
|  |  | 21. GSM38208 | Skeletal muscle, adrenalectomized mice | 10 | 67 |
|  |  | 22. GSM38113 | Skeletal muscle, adrenalectomized mice | 7 | 46 |
|  |  | 23. GSM5435 | Testis, busulphan-treated | 3 | 36 |
|  | LSAGE | 1. GSM34020, GSM45132 | CNS | 17 | 121 |
|  |  | 2. GSM34021, GSM45133 | CNS | 33 | 103 |
|  |  | 3. GSM34034 | CNS | 9 | 101 |
|  |  | 4. GSM33991, GSM45141 | CNS | 12 | 98 |
|  |  | 5. GSM45165 | CNS | 3 | 26 |
|  |  | 6. GSM34030 | CNS | 2 | 18 |
|  |  | 7. GSM34004 | CNS | 2 | 18 |

^a^, tag counts; ^b^, tags per million. CNS, the central nervous system; LSAGE, long serial analysis of gene expression; SAGE, serial analysis of gene expression.
